# Real‐world evidence that among atrial fibrillation patients warfarin is associated with reduced nonelective admissions compared with those on DOACs

**DOI:** 10.1002/clc.24146

**Published:** 2023-09-08

**Authors:** Dahai Yu, Julian Brown, W. David Strain, David Simmons

**Affiliations:** ^1^ Primary Care Centre Versus Arthritis, School of Medicine, Faculty of Medicine & Health Sciences, Keele University Keele UK; ^2^ Swaffham Downham Primary Care Network, NHS UK; ^3^ Diabetes and Vascular Medicine Research Centre, Institute of Biomedical and Clinical Science and College of Medicine and Health, University of Exeter Exeter UK; ^4^ Macarthur Clinical School, School of Medicine Western Sydney University Sydney New South Wales Australia

**Keywords:** bleeding, direct‐acting oral anticoagulants, ED, hospitalization, tapered matching, warfarin

## Abstract

**Background:**

Randomized trials show inconsistent estimates on risks of direct‐acting oral anticoagulants (DOACs) versus warfarin in bleeding and mortality for atrial fibrillation (AF) patients. Trials are confounded by additional DOAC adherence support, while warfarin has a low time in therapeutic range. Few real‐world studies compared emergency hospitalization risk between DOAC and warfarin users in AF. This study aimed to determine emergency hospitalization risk for AF patients on DOACs or warfarin in real‐world settings.

**Methods:**

A tapered‐matched real‐world cohort extracted data from 412 English general practices' primary care records linked with emergency department (ED) and hospitalization data from the ECLIPSE database. AF patients with new DOAC or warfarin prescriptions were included. The primary outcome was all‐cause ED attendance; the secondary outcomes were ED re‐attendance, nonelective hospitalization, and rehospitalization within 12 months. Weighted Cox regression estimated relative risk difference.

**Results:**

39 201 DOAC patients were matched with 13 145 warfarin patients. DOAC patients had a 25% higher likelihood of attending ED (odds ratio 1.25; 95% confidence interval [CI] 1.01–1.55). DOAC use also associated with higher ED re‐attendance, nonelective hospitalization, and rehospitalization within 12 months: 1.41 (95% CI 1.00–1.98), 1.26 (1.00–1.57), and 1.54 (1.01–2.34), respectively, with *p*‐values < .05.

**Conclusions:**

DOACs for AF thromboprophylaxis are associated with the increased risk of ED attendance, recurrent hospitalization, and numerical rise in ED re‐attendance and first nonelective hospitalization compared to warfarin. However, these real‐world data cannot establish if this difference results from medication adherence, lack of regular DOAC clinic monitoring, unmeasured confounders, or fundamental agent efficacy disparities.

## Graphical abstract

Real‐world study comparing direct‐acting oral anticoagulants (DOACs) and warfarin for atrial fibrillation patients. DOACs are associated with increased emergency department attendance, hospitalizations, and re‐hospitalizations within 12 months compared to warfarin. Differences may result from adherence, monitoring, or unmeasured factors.

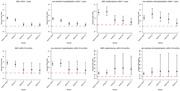

## 1 INTRODUCTION

It has been estimated over 1.2 million people in the UK have been diagnosed with atrial fibrillation (AF) and thousands remain undiagnosed.^1^ Compared with those without AF, people with AF have an estimated three to five times higher risk of ischemic stroke. Up to 30% of AF patients are admitted to hospital due to ischemic stroke with a higher risk of fatal outcome.^2^ The risk of AF‐related stroke is attenuated by anticoagulation, such that stroke rates of people with AF and adequate anticoagulant are close to those without recorded AF.^3^

Warfarin has been the anticoagulant of choice for >60 years. When appropriately dosed, to an international normalized ratio (INR) of between 2 and 3 these are very effective, however when the time in the therapeutic range falls below 65%, these agents provide negligible benefit when compared with aspirin, which itself is not superior to placebo in disease prevention. The utilization of direct‐acting oral anticoagulants (DOACs), including dabigatran, rivaroxaban, and apixaban, has risen rapidly, as they reduced laboratory and primary care attendance. Central organizations such as the Royal College of General Practitioners issued guidance encouraging widespread shifting from warfarin to the DOACs.^4^ Their use has surpassed the use of warfarin due to their fixed dose and lower requirements for monitoring^5^ and multiple studies inferring superior safety data.^6^

DOACs are now the most expensive medications utilized in primary care prescribing with procurement costs approaching £1 billion per annum.^4^

ECLIPSE (Equity of Care Insights for Patient Safety & Engagement) is a centralized database provided for the NHS to provide risk stratification longitudinally tracking data from over 20 million patients to provide prescribing safety alerts for GPs in England. The system contains over 10 billion rows of patient data updated each week and is centrally contracted by NHS Digital to provide safety algorithms for over 2500 GP surgeries.^7^

It has a central risk stratification system called PRISM (Patient Risk Identification through Statistical Modeling). This system runs a mixture of iterative calculations and machine learning to track patient outcomes in relation to phenotypes, monitoring, and medications. It claims to automatically adjust for over 2000 confounding parameters and runs detailed analytics against all medications listed as requiring enhanced monitoring in primary care.^8^

DOACs are one of the groups within this group, and it is suggested that all patients on these agents have three monthly reviews.^9^ Eclipse reports that monitoring levels within primary care were less than 50% compliant with the current guidelines during the evaluation period and undertook enhanced safety analytics to assess the impact. The system reported finding a 1.42 (confidence interval [CI] 1.14–1.60) fold risk of nonelective admissions for AF patients on DOACs compared with those on warfarin.

This study was designed to compare the risks of attendance at the emergency department (ED) and nonelective hospitalization in the first 12 months of DOAC and warfarin prescription in people with AF. We have used a recently developed statistical approach: tapered multivariable matching.^10^, ^11^ This allows us to examine the extent of the observed difference in outcomes between DOAC and warfarin users and more importantly, to investigate how potential confounders relate to the risk differentiation. In tapered matching, we sequentially match the DOAC user group to the warfarin user group with an increasingly comprehensive set of variables. As we incrementally match the DOAC user group and the warfarin user group, we can directly observe how the matched cohorts change both in terms of risk of outcome and in terms of unmatched covariables.

## 2 METHODS

### 2.1 Data sources

A large UK primary care electronic health record database from eight participating clinical commissioning groups (CCGs) in England covering 412 practices was used for this study.^12^ All included practices were linked at the patient level to hospital admission data (Secondary User Service [SUS] data). The covariates were defined by Read codes recorded in the electronic health records at primary care settings^13^, ^14^ and the outcomes were defined by International Classification of Diseases, 10th revision codes recorded in SUS data (codes list is accessible by reasonable request via corresponding author).^15^, ^16^ The use of this database in this manner was approved by the South West ‐ Exeter Research Ethics Committee (REC reference: 17/SW/0001).

The study period ran from April 1, 2017 to December 31, 2018. All patients with AF newly prescribed the oral anticoagulants warfarin, dabigatran, rivaroxaban, or apixaban, and aged from 18 to 99 years at the date of study entry, were eligible for enrollment. The entry date was defined as the date of the first prescription of any of the anticoagulant drugs. To facilitate a direct comparison between new users of DOACs against new users of warfarin, and to reduce the impact of indication bias, patients were excluded if they had any anticoagulant prescription in the prior 12 months before the entry date. To ensure the quality of data, patients were also excluded if they had less than 12 months of registration history before entry.

Our primary outcome was any attendance to ED within the first 12 months of anticoagulant prescription. Secondary outcomes of interest included: (i) any incident nonelective hospitalization within the 12 months following the first anticoagulant prescription; (ii) nonelective re‐hospitalization (having ≥2 nonelective hospitalizations) within the 12 months since the first anticoagulant prescription; (iii) ED re‐attendance (having ≥2 ED events) within 12 months since the first anticoagulant prescription; (iv) any incident nonelective hospitalization within the 6 months following the first anticoagulant prescription; (v) any incident ED event within the 6 months since the first anticoagulant prescription; (vi) re‐nonelective hospitalization (having ≥2 nonelective hospitalizations) within the 6 months since the first anticoagulant prescription; and (vii) ED re‐attendance (having ≥2 ED events) within 6 months since the first anticoagulant prescription.

Patients were followed from their first prescription of an anticoagulant until they experienced an outcome of interest or by completion of 12 months (or 6 months) without an event. Patients were excluded if they stopped or suspended treatment up to 30 days after the first anticoagulant prescription. Patients were excluded if they switched between warfarin and DOAC or vice versa within the first 12 months.

### 2.2 Statistical methods

#### 2.2.1 Matching method

This study used a tapered matching method to generate a series of matches for each comparison of DOAC and warfarin.^17^ For the DOAC user group, we performed six matches that constructed sets of pairs of warfarin users as shown in Supporting Information: Figure 1. First, demographic factors match paired patients by their age at incident anticoagulant prescription, and gender. Second, the DOAC user group was match‐controlled (warfarin user group) for all demographic factors and clinical measurements (body mass index and systolic blood pressure). Third, the DOAC user group were matched with the warfarin user group for all variables in the first two matches as well as routine blood test results (international normalization ratio [INR], HbA1c, and total cholesterol). Fourth, the DOAC user group were matched with the warfarin user group for all variables in the first three matches as well as prior bleeding or replated comorbidities (any bleeding, upper GI bleeding, hematuria, haemoptysis, chronic renal disease, alcohol dependence, chronic liver disease or pancreatitis, chronic obstructive pulmonary disease, dyspepsia, esophageal varices, peptic ulcer, knee/hip surgery, and hip fracture) and cardiovascular disease (CVD) subtypes (valvular heart disease, congestive cardiac failure, coronary heart disease, venous thromboembolism, ischemic stroke/TIA, diabetes, and hypertension). Fifth, the DOAC user group was matched with the warfarin user group for all variables in the first four matches as well as prescriptions potentially relevant to bleeding event (antibiotics, anticonvulsants, corticosteroid, antidepressant, antiplatelet, NSAIDS, and proton pump inhibitor) and prescriptions relevant to management of CVD (antihypertensive, antidiabetes, and statin). Matched variables were defined by established Read codelists (codelists available from www.keele.ac.uk/mrr).

In each step of matching, we used a coarsened exact matching (CEM) algorithm involving a monotonic imbalance reducing matching method, which means that the balance between the treated and control groups is chosen by ex‐ante user choice rather than discovered through the usual laborious process of “checking after the fact, tweaking the method, and repeatedly re‐estimating.”^18^ Patients in both the DOAC and warfarin user groups matched on Step‐5 were retained (Supporting Information: Figure 1).

Via CEM we restricted the comparison of DOAC and warfarin user groups to areas of common support, that is, sufficient overlap between the two groups, on the above key factors in the proton pump inhibitor five‐steps, coarsened using the default Sturges measure of bin size.^19^ After excluding patients (Supporting Information: Figure 1) who were off common support, we then used Entropy Balancing^20^ to efficiently minimize differences in the distribution of matching variables between DOAC and warfarin user groups. Entropy balancing involves maximum entropy reweighting the matched sample in each matching step to key target moments (mean, variance, and skewness). For continuous matching variables, all three moments should be met; for binary variables, the only target moment is the mean as it is only sufficient to match higher moments (variance and skewness).

Weighted logistic regression, incorporating matching weights estimated from each matching step by entropy matching, was applied in each matching step. This provided an estimate of the association between DOAC use and risks of outcomes, with warfarin as the reference group.^20^ Data on each variable were missing in <6% of eligible cohort members. Based on the worst scenario of 5% of patients with ≥1 missing data, five imputed data sets were created for multiple imputations with chained equations, and estimations were made by Robin's rule.^21^ The analyses were conducted using Stata/MP, version 17.0 (StataCorp LLC). Statistical significance was set at two‐tailed *p* < .05.

## 3 RESULTS

**Figure 1:**
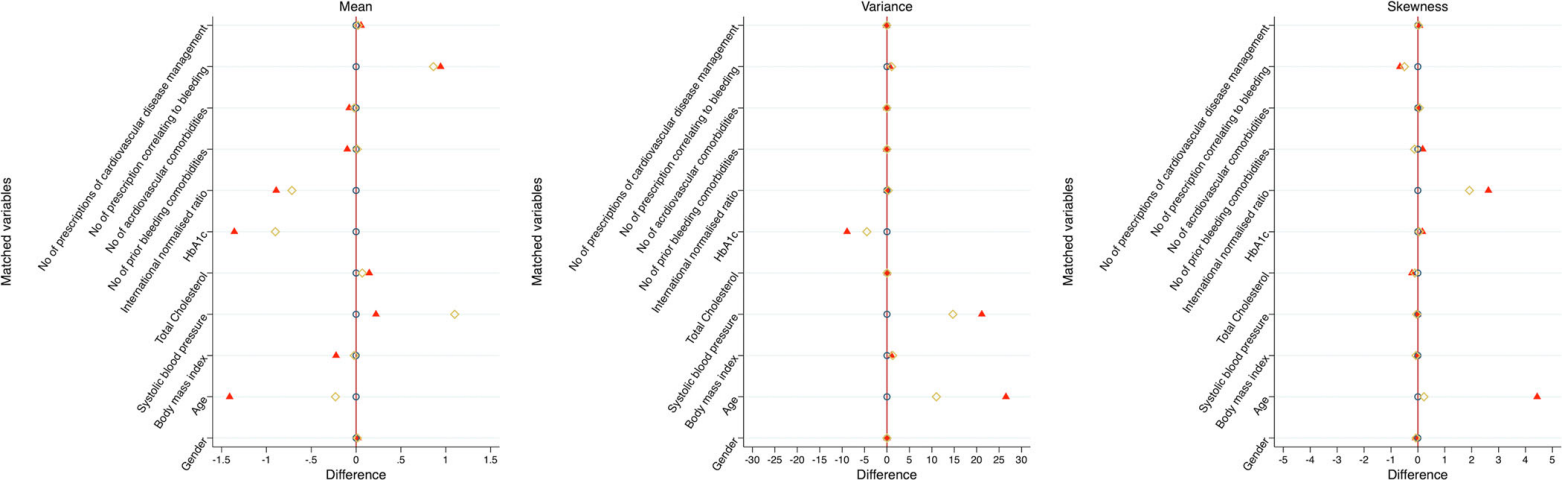
Distribution of difference of means, variance, and skewness on matched variables in the unmatched and matched cohorts. Triangles indicate measurements from unmatched cohorts; diamonds indicate measurements from coarsened exact matching; circles indicate measurements from entropy‐matching cohorts.

We identified 41 755 people with AF and with incident DOAC prescription and 13 369 people with AF with incident warfarin prescription between 2017 and 2018. Table 1 and Figure 1 demonstrate that the matched variables were quite different between DOAC users and warfarin users. As expected, DOAC users had a lower INR than those on Warfarin. Those prescribed DOACs also had a past medical history demonstrating fewer prior bleeding events or CVD comorbidities compared with warfarin users, suggesting there was a propensity to use DOACs in individuals with a lower risk of complications.

**Table 1:** Baseline characteristics in the comparison cohorts.

	Unmatched cohorts		
	Incident DOAC users (*N* = 41 755)	Incident warfarin users (*N* = 13 369)	*p*‐Value for matching variables
Age, years	74.4 (10.8)	75.8 (9.5)	<.0001
Male gender, *n* (%)	23 685 (56.7)	7844 (58.7)	<.0001
Body mass index, kg/m^2^	29.0 (6.3)	29.2 (6.2)	.003
Systolic blood pressure, mmHg	132 (17)	132 (16)	.231
High‐density lipoprotein cholesterol, mmol/L	1.4 (0.4)	1.4 (0.4)	<.0001
Low‐density lipoprotein cholesterol, mmol/L	2.5 (1.0)	2.4 (1.0)	<.0001
Triglyceride, mmol/L	1.4 (0.8)	1.5 (0.8)	<.0001
Total cholesterol, mmol/L	4.5 (1.1)	4.4 (1.1)	<.0001
INR	1.4 (0.7)	2.3 (0.7)	<.0001
HbA1c, mmol/mol/%	42.4 (9.2)	43.8 (9.6)	<.0001
No of prior CVD or bleeding comorbidities^a^			
0	8405 (20.1)	2284 (17.1)	<.0001
1	13 829 (33.1)	4053 (30.3)	
2	10 731 (25.7)	3603 (27.0)	
3	5571 (13.3)	2118 (15.8)	
4	2247 (5.4)	851 (6.4)	
5	710 (1.7)	303 (2.3)	
6	213 (0.5)	126 (0.9)	
7	35 (0.1)	24 (0.2)	
8	12 (0.0)	4 (0.0)	
9	2 (0.0)	3 (0.0)	
No of prescriptions potentially correlating with bleeding/CVD^b^			
0	1350 (3.2)	1079 (8.1)	<.0001
1	4720 (11.3)	2469 (18.5)	
2	6107 (14.6)	3005 (22.5)	
3	6814 (16.3)	2394 (17.9)	
4	7076 (17.0)	1975 (14.8)	
5	6512 (15.6)	1276 (9.5)	
6	4988 (12.0)	698 (5.2)	
7	2784 (6.7)	321 (2.4)	
8	1114 (2.7)	114 (0.9)	
9	263 (0.6)	36 (0.3)	
10	27 (0.1)	2 (0.01)	

Abbreviations: DOACs, direct‐acting oral anticoagulants; GI, gastrointestinal; INR, international normalized ratio; NSAIDS, nonsteroidal anti‐inflammatory drugs.

^a^ Indicates prior bleeding or related comorbidities (any bleeding, upper GI bleeding, hematuria, hemoptysis, chronic renal disease, cancer, alcohol dependence, chronic liver disease or pancreatitis, chronic obstructive pulmonary disease, dyspepsia, esophageal varices, peptic ulcer, knee/hip surgery, and hip fracture) and cardiovascular disease (CVD) subtypes (valvular heart disease, congestive cardiac failure, coronary heart disease, venous thromboembolism, ischemic stroke/tia, diabetes, hypertension).

^b^ Indicates prescriptions relevant to bleeding (antibiotics, anticonvulsants, corticosteroid, antidepressant, antiplatelet, NSAIDS, and proton pump inhibitors) and prescription relevant to management cardiovascular diseases (antihypertensive, antidiabetes, and statin).

By coarsened exact matching, 39 201 incident DOAC users with AF were matched with 13 145 warfarin users with AF via five matching steps (Supporting Information: Figure 1). After coarsened exact matching, the matched variables tended to be closer (Table 1, Figure 1, and Table 2). In particular, matched variables were very similar after samples were weighted by entropy matching (Figure 1 and Table 2) in terms of mean, variance, and skewness.

**Table 2:** Each comorbidity and prescription distribution in the unmatched and matched cohorts.

	Unmatched cohorts			Cohorts after coarsened exact matching			Cohorts after entropy matching		
	Incident DOAC users (*N* = 41 755)	Incident warfarin users (*N* = 13 369)	*p*‐Value	Incident DOAC users (*N* = 39 201)	Incident warfarin users (*N* = 13 345)	*p*‐Value	Incident DOAC users (*N* = 39 201)	Incident warfarin users (*N* = 13 345)	*p*‐Value
Antibiotics	19 232 (46.1)	2503 (18.7)	<.0001	18 066 (46.1)	2451 (18.7)	<.0001	36.3 (0.01)	33.4 (0.02)	.171
Anticonvulsants	3552 (8.5)	777 (5.8)	<.0001	3310 (8.4)	764 (5.8)	<.0001	8.2 (0.01)	10.5 (0.01)	.087
Corticosteroid	5139 (12.3)	749 (5.6)	<.0001	4802 (12.3)	737 (5.6)	<.0001	9.6 (0.01)	10.1 (0.01)	.695
Antidepressant	8294 (19.9)	1783 (13.3)	<.0001	7811 (17.9)	1756 (13.4)	<.0001	19.7 (0.01)	21.0 (0.01)	.445
Antiplatelet	14 211 (34.0)	1794 (13.4)	<.0001	13 398 (34.2)	1768 (13.5)	<.0001	31.9 (0.01)	28.0 (0.02)	.055
NSAIDS	24 849 (59.5)	5721 (42.8)	<.0001	23 273 (59.4)	5642 (42.9)	<.0001	58.1 (0.01)	59.2 (0.01)	.556
Proton pump inhibitors	18 536 (44.4)	4111 (30.8)	<.0001	17 336 (44.2)	4058 (30.9)	<.0001	44.2 (0.01)	45.8 (0.02)	.442
Antihypertensive	36 893 (88.4)	11 250 (84.2)	<.0001	34 728 (88.6)	11071 (84.2)	<.0001	88.7 (0.01)	88.3 (0.01)	.743
Antidiabetes	5669 (13.6)	1877 (14.0)	.175	5567 (14.2)	1859 (14.1)	.867	21.4 (0.01)	20.9 (0.01)	.796
Statin	22 830 (54.7)	7067 (52.9)	<.0001	21491 (54.8)	6948 (52.9)	<.0001	59.8 (0.01)	60.6 (0.02)	.680
Knee/hip surgery	1197 (2.9)	379 (2.8)	.863	1118 (2.9)	374 (2.9)	.968	4.4 (0.01)	4.7 (0.01)	.771
Hip fracture	281 (0.7)	79 (0.6)	.305	265 (0.7)	78 (0.6)	.310	0.8 (0.002)	1.4 (0.004)	.138
Any bleeding	9244 (22.1)	3528 (26.4)	<.0001	8564 (21.9)	3447 (26.2)	<.0001	36.6 (0.01)	37.5 (0.02)	.629
Upper GI bleeding	642 (1.5)	176 (1.3)	.066	591 (1.5)	173 (1.3)	.113	2.1 (0.004)	2.0 (0.004)	.866
Hematuria	3145 (7.5)	1400 (10.5)	<.0001	2927 (7.5)	1365 (10.4)	<.0001	11.8 (0.01)	11.7 (0.01)	.967
Haemoptysis	351 (0.8)	141 (1.1)	.022	325 (0.8)	135 (1.0)	.035	1.8 (0.003)	2.4 (0.005)	.270
Chronic renal disease	2519 (6.0)	1160 (8.7)	<.0001	2329 (5.9)	1121 (8.5)	<.0001	8.5 (0.01)	7.9 (0.007)	.567
Cancer	2445 (5.9)	858 (6.4)	.017	2246 (5.7)	832 (6.3)	.011	9.3 (0.01)	8.7 (0.01)	.609
Alcohol dependence	140 (0.3)	30 (0.2)	.044	130 (0.3)	30 (0.2)	.063	0.5 (0.002)	0.4 (0.002)	.534
Chronic liver disease or pancreatitis	301 (0.7)	90 (0.7)	.805	278 (0.7)	88 (0.7)	.893	0.8 (0.001)	1.1 (0.001)	.612
Chronic obstructive pulmonary disease	3035 (7.3)	945 (7.1)	.437	2904 (7.4)	933 (7.1)	.238	10.3 (0.01)	9.3 (0.01)	.426
Congestive cardiac failure	1755 (4.2)	746 (5.6)	<.0001	1666 (4.3)	739 (5.6)	<.0001	6.2 (0.01)	7.4 (0.01)	.224
Coronary heart disease	1826 (4.4)	619 (4.6)	.209	1775 (4.5)	614 (4.7)	.497	7.7 (0.01)	7.3 (0.01)	.730
Diabetes	5940 (14.2)	2126 (15.9)	<.0001	5813 (14.8)	2114 (16.1)	.001	25.0 (0.01)	24.8 (0.01)	.889
Dyspepsia	511 (1.2)	160 (1.2)	.897	460 (1.2)	154 (1.2)	.986	4.0 (0.005)	3.0 (0.006)	.232
Hypertension	23 326 (55.9)	7713 (57.7)	<.0001	22 380 (57.1)	7625 (58.0)	.066	68.1 (0.01)	68.7 (0.02)	.724
Ischemic stroke/TIA	6952 (16.7)	2302 (17.2)	.125	6667 (17.0)	2279 (17.3)	.384	20.1 (0.01)	19.1 (0.01)	.495
Esophageal varices	24 (0.1)	6 (0.04)	.587	21 (0.1)	5 (0.04)	.489	0.1 (0.008)	0.02 (0.002)	.165
Peptic ulcer	365 (0.9)	80 (0.6)	.002	343 (0.9)	80 (0.6)	.003	0.9 (0.002)	0.6 (0.002)	.391
Valvular heart disease	1047 (2.5)	515 (3.9)	<.0001	987 (2.5)	510 (3.9)	<.0001	3.3 (0.004)	3.9 (0.01)	.070
Venous thromboembolism	1133 (2.7)	462 (3.5)	<.0001	1081 (2.8)	457 (3.5)	<.0001	5.2 (0.01)	5.4 (0.01)	.114

*Note*: Weighted estimation was presented for cohorts after entropy matching.

Abbreviations: DOACs, direct‐acting oral anticoagulants; NSAIDS, nonsteroidal anti‐inflammatory drugs; TIA, transient ischemic attack.

The rates of each outcome in unmatched cohorts and matched cohorts for incident DOAC users and incident warfarin users are presented in Table 3. Higher rates of outcomes were identified among incident DOAC users.

**Table 3:** The rates of outcomes in the comparison cohorts.

	Unmatched cohorts		Cohorts after coarsened exact matching	
	Incident DOAC user (*N* = 41 755)	Incident warfarin (*N* = 13 369)	Incident DOAC user (*N* = 39 201)	Incident warfarin (*N* = 13 145)
All‐cause nonelective hospitalization within 6‐month, *n* (%)	2111 (5.06)	257 (1.92)	2021 (5.16)	251 (1.91)
All‐cause nonelective hospitalization within 1‐year, *n* (%)	3869 (9.27)	545 (4.08)	3707 (9.46)	538 (4.09)
All‐cause nonelective rehospitalization within 6‐month, *n* (%)	595 (1.42)	70 (0.52)	566 (1.44)	68 (0.52)
All‐cause nonelective rehospitalization within 1‐year, *n* (%)	1345 (3.22)	173 (1.29)	1281 (3.27)	169 (1.29)
All‐cause ED admission within 6‐month, *n* (%)	2838 (6.80)	331 (2.48)	2724 (6.95)	324 (2.46)
All‐cause ED admission within 1‐year, *n* (%)	5425 (12.99)	754 (5.64)	5194 (13.25)	743 (5.65)
All‐cause ED admission reattendance within 6‐month, *n* (%)	926 (2.22)	95 (0.71)	893 (2.28)	94 (0.72)
All‐cause ED admission within 1‐year, *n* (%)	2127 (5.09)	243 (1.82)	2035 (5.19)	239 (1.82)

Abbreviations: DOACs, direct‐acting oral anticoagulants; ED, emergency department.

**Figure 2:**
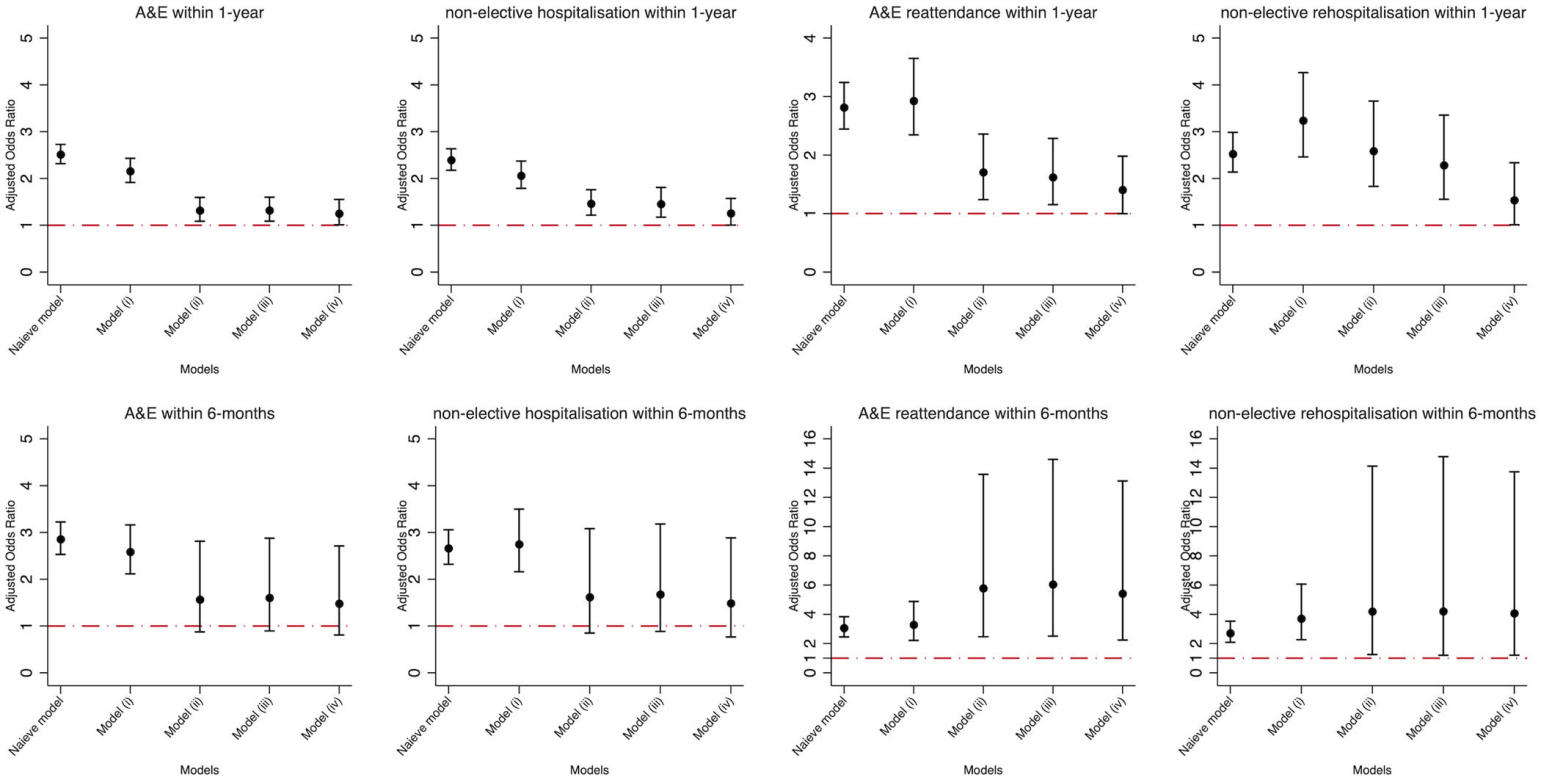
Adjusted odds ratios for association between DOAC (reference to warfarin) and risks of all‐cause nonelective (re)hospitalization and all‐cause ED reattendance. Naive model weighted for age at incident anticoagulant prescription, gender; model (i) weighted for all adjusted variables in model naïve model plus body mass index, systolic blood pressure; model (ii) weighted for all adjusted variables in model (i) plus total cholesterol, HbA1c, international normalized ratio; model (iii) weighted for all adjusted variables in model (ii) plus prior bleeding or related comorbidities (any bleeding, upper GI bleeding, hematuria, haemoptysis, chronic renal disease, cancer, alcohol dependence, chronic liver disease or pancreatitis, chronic obstructive pulmonary disease, dyspepsia, esophageal varices, peptic ulcer, knee/hip surgery, and hip fracture) and cardiovascular disease subtypes (valvular heart disease, congestive cardiac failure, coronary heart disease, venous thromboembolism, ischemic stroke/TIA, diabetes, and hypertension); model (iv) weighted for all adjusted variables in model (iii) plus prescriptions relevant to bleeding (antibiotics, anticonvulsants, corticosteroid, antidepressant, antiplatelet, NSAIDS, and proton pump inhibitors) and prescription relevant to management cardiovascular diseases (antihypertensive, antidiabetes, and statin). DOACs, direct‐acting oral anticoagulants; ED, emergency department; GI, gastrointestinal; NSAIDS, nonsteroidal anti‐inflammatory drugs; TIA, transient ischemic attack.

Compared with warfarin users, DOAC users were associated with an increased risk of ED, nonelective hospitalization, ED re‐attendance, and nonelective rehospitalization within 12 months after the first anticoagulant prescription (Figure 2). The odds ratio weighted by matched variables in each matching step was 2.51 (95% CI: 2.31–2.73), 2.39 (2.18–2.63), 2.81 (2.44–3.24), and 2.53 (2.14–2.99) for ED, nonelective hospitalization, ED re‐attendance and nonelective rehospitalization within 12 months from naive model weighted for age at incident anticoagulant prescription and gender; 2.16 (1.91–2.43), 2.06 (1.79–2.37), 2.93 (2.34–3.65), and 3.24 (2.46–4.26) for model (i) weighted for all matched variables in naive model plus body mass index and systolic blood pressure; 1.32 (1.09–1.59), 1.46 (1.22–1.76), 1.71 (1.24–2.36), and 2.59 (1.83–3.65) for model (ii) weighted for all matched variables in model (i) plus routine blood test results (INR, HbA1c, and total cholesterol); 1.32 (1.09–1.60), 1.46 (1.17–1.81), 1.62 (1.15–2.28), and 2.28 (1.56–3.35) for model (iii) weighted for all matched variables in model (ii) plus prior bleeding or relevant comorbidities (any bleeding, upper GI bleeding, hematuria, haemotysis, chronic renal disease, alcohol dependence, chronic liver disease or pancreatitis, chronic obstructive pulmonary disease, dyspepsia, esophageal varices, peptic ulcer, knee/hip surgery, and hip fracture) and CVD subtypes (valvular heart disease, congestive cardiac failure, coronary heart disease, venous thromboembolism, ischemic stroke/TIA, diabetes, and hypertension); 1.25 (1.01–1.55), 1.26 (1.00–1.57), 1.41 (1.00–1.98), and 1.54 (1.01–2.34) for model (iv) weighted for all matched variables in model (iii) plus prescriptions potentially relevant to bleeding event (antibiotics, anticonvulsants, corticosteroid, antidepressant, antiplatelet, NSAIDS, and proton pump inhibitor) and prescriptions relevant to management of CVD (antihypertensive, antidiabetes, and statin).

Compared with warfarin users, DOAC users were associated with an increased risk of ED, nonelective hospitalization, re‐ED, and re‐nonelective hospitalization within 6 months since the first anticoagulant prescription (Figure 2). For models (i), (ii), (iii), (iv), and (v), risk of ED, nonelective hospitalization, ED re‐attendance, and nonelective rehospitalization within 6 months, the odds ratio was 2.86 (2.53–3.22), 2.59 (2.12–3.16), 1.57 (0.87–2.81), 1.60 (0.90–2.88), and 1.48 (0.81–2.71) for risk of ED; 2.66 (2.32–3.06), 2.75 (2.16–3.50), 1.62 (0.85–3.08), 1.68 (0.88–3.18), and 1.49 (0.77–2.88) for nonelective hospitalization; 3.07 (2.46–3.83), 3.29 (2.22–4.88), 5.78 (2.46–13.57), 6.05 (2.51–14.59), and 5.42 (2.24–13.12) for ED reattendance; 2.71 (2.08–3.53), 3.71 (2.26–6.07), 4.20 (1.25–14.13), 4.21 (1.20–14.78), and 4.07 (1.21–13.75) for nonelective rehospitalization, respectively.

## 4 DISCUSSION

We have demonstrated that in a real‐world setting, people requiring thromboprophylaxis for AF and treated with DOACs had an increased risk in ED re‐attendance, nonelective hospitalization, and rehospitalization over the first 12 months compared to an initial prescription of warfarin. This was true for both initial attendance to ED, recurrent visits to ED, and recurrent unplanned hospitalizations. This is contrary to the reports of randomized controlled trials, that suggest DOACs should be associated with a lower risk of either thromboembolic events, serious adverse bleeding events, or both. This was based on an analysis of routinely collected primary care electronic health records linked with hospitalization data in a population in the UK healthcare system.

Our results contrast the outcomes of the randomized controlled clinical trials. A recent network meta‐analysis of these RCTs showed that DOACs are safer than warfarin in relation to major and intracranial bleeding.^22^ However, interestingly, the same analysis presented a higher risk of gastrointestinal bleeding with dabigatran, edoxaban, and rivaroxaban than with warfarin. In addition, edoxaban (30 mg and 60 mg two times per day) significantly increased the risk of clinically relevant bleeding compared with warfarin.^22^ The RCTs have not always demonstrated clear‐cut benefits. Two studies have reported an elevated risk of mortality with lower doses of apixaban and rivaroxaban compared with warfarin.^23^, ^24^ Another Danish study showed decreased mortality for apixaban,^25^ but QResearch showed equivalent risk to warfarin for such patients. For the outcome of ischemic stroke, both the Danish study and QResearch showed DOACs were equivalent to warfarin.^25^

There are several potential explanations for our observations of increased healthcare utilization amongst DOAC users. Amongst these include a difference in the adherence to the drugs in a real‐world setting, a relative difference in the efficacy of these agents in the UK healthcare environment, or simple residual confounding in the populations compared.

As DOACs do not require routine blood testing, a burden on both patients and prescribers, they have been increasingly prescribed to replace the traditional anticoagulant, warfarin. However, with that regular blood test comes a process to reinforce the importance of good adherence. Adherence and persistence with DOACs, however, do not achieve the same standard in a real‐world setting,^26^ one database study suggested adherence of DOACs runs at approximately 63.1%, 64.7%, and 66.5% for rivaroxaban, apixaban, and dabigatran over a year, respectively. This low rate of adherence is not seen in clinical trials where regular reminders to follow protocol. With the rate of nonadherence running at approximately one‐third this could account for the increased rate of ED attendances and unplanned hospital admissions in those treated with DOACs compared to those treated with warfarin.

Respective drug adherence could also account for a potential difference in efficacy of the drugs. All of the landmark trials comparing DOACs and warfarin, based predominantly in Europe and parts of the United States, have poor time in the therapeutic range (TTR) of warfarin. The mean TTR in the RE‐LY study (dabigatran), ARISTOTLE (apixaban), and ROCKET AF (rivaroxaban) were 64%,^27^ 62.2%,^28^ and 55%,^29^ respectively, suggesting that none of these trials met the minimum acceptable standard established by the National Institute of Clinical and Healthcare Excellence.^30^ A TTR of less than 65% has been suggested to be no more effective than simple antiplatelets in preventing events in those with nonvalvular AF.^31^ Further, aspirin alone is little better than placebo at preventing stroke, indeed in some studies has been suggested to increase complications without affording benefit. In the UK, the average TTR ranges between 71% for those monitored in primary care to 78% for those attending secondary care clinics.^32^, ^33^ The TTR for the warfarin cohort in the data set utilized for this study was estimated to be 70.8%. This estimation was derived from the monthly percentage of patients whose INR was between 2.0 and 3.0.

A subgroup analysis of the RE‐LY study compared outcomes from those treated with dabigatran and those treated with warfarin stratified by the mean TTR in each center. It demonstrated incremental reductions in benefit, as TTR increased such that there were clear advantages from DOAC when TTR was <65% (event rate was 7.9%, or 9.7% and for Warfarin for TTR 57%–65% or <57%, respectively, compared with 7.0% and 6.7% for dabigatran 150 mg in the same centers). However, when TTR was greater than 65%, such as we see in the UK routine practice warfarin was superior to DOAC at both preventing events and avoiding adverse effects (event rate 6.6% and 6.4% for TTR 66%–72% and >72%, respectively, compared with 7.2% and 6.8% in the same centers).^34^ Given that these latter TTR values are more in keeping with usual practice in the UK, it is should not come as a surprise that warfarin was superior to DOAC at reducing ED admissions and unplanned hospitalizations. It remains possible that this is a product of residual confounding, however, this is unlikely.

Previous cohort studies, based on real‐world data, have compared the risk of different outcomes between patients treated with DOACs and those treated with warfarin.^35^, ^36^, ^37^, ^38^ For instance, a US cohort extracted from a commercial healthcare database found that patients with valvular AF who were new users of DOACs had lower risks of ischemic stroke or systemic embolism and major bleeding compared with new users of warfarin.^35^ Similarly, a Japanese cohort extracted from an AF registry found that warfarin and DOACs exhibited equivalent 3‐year stroke and all‐cause mortality rates, but DOACs showed a reduced risk of major bleeding.^38^ In a Norwegian national AF registry cohort, it was revealed that all DOACs were similarly effective as warfarin in preventing ischemic stroke, TIA, or systemic embolism, with similar safety in terms of bleeding.^36^ Another US cohort based on US Centers for Medicare & Medicaid Services data found that all DOACs had a lower risk of major adverse cardiac events compared to warfarin.^37^ However, it is important to note that the outcomes explored in previous studies differ from those in the current study, as the current study focused on the short‐term risk of ED visits and nonelective (re)hospitalizations. Additionally, discrepancies in study findings may also be attributable to differences in population profiles (e.g., ethnicity, age, and gender) and the validity of electronic health records (e.g., misclassifications in the definition of covariates and outcomes due to different coding systems^39^). Furthermore, the variation in adherence to DOACs across different studies could also contribute to the discrepancy in the findings. Future validation studies using cohorts with similar population structures and adjusting for adherence to DOACs are warranted.

A key strength of this work was the application of a novel, tapered matching method to form a ‘quasi‐trial’ sample to compare the risk of hospitalization for the three outcomes between incident DOAC and warfarin users with AF. Through tapered matching, we were able to transparently examine how differences in specific sets of confounders contributed to the risk of ED and nonelective hospitalization. By sequentially matching and weighting for differences in demographic characteristics, clinical measurements, prior bleeding and relevant comorbidities, CVD, prescriptions potentially relevant to bleeding, and CVD management, we were able to compare the risks of outcomes after each match between new DOAC and the new warfarin users. This, in turn, allowed the importance of potential confounders (e.g., perceived risks) to be assessed and adjusted for. This prospective cohort incorporating patients with AF was derived from a large primary care database in England that was linked with hospitalization data which have been shown to be of good quality in terms of representativeness, coverage, validity, and consistency in records of comorbidities and prescriptions.^40^ Hospitalization data used in the study were complete as SUS data captures all hospitalization information for patients and its recorded outcomes, which has also been proven to have good validity, including those experiencing events outside of the CCG catchment.^41^ There are some limitations in this study. First, as the sample size was restricted, instead of exactly matching each relevant bleeding and CVD comorbidity and prescription, we have matched the number of bleeding and CVD comorbidities and associated prescriptions. However, we have compared the bleeding and CVD comorbidities, each antihypertensive and each antidiabetes prescription after matching with no significant difference identified between DOAC and warfarin user groups (Table 2). Second, the time‐varying/cumulative effects on outcomes between DOAC and warfarin were not examined in this study and should be examined in future studies. Third, death linkage was not accessible for this study, therefore, the competing risk of death could not be evaluated. DOACs are a heterogeneous group of drugs with different mechanisms of action, dosing, pharmacokinetics, efficacy, and safety.^42^ Comparing each DOAC with warfarin concerning the risk of outcomes is therefore important. However, due to current data access restrictions, a direct comparison between each DOAC and warfarin regarding outcome risks was not conducted in the current study. Future studies should include such comparisons. While understanding the cause of an ED visit/admission is important, it's worth noting that the recorded cause for the occurrence of such visits/admissions might not always correspond to the final diagnosis. Unfortunately, due to constraints in data accessibility, performing a cause‐specific analysis was not viable within the scope of this current study. Future validation studies with cause‐specific outcomes are warranted.

### 4.1 Clinical implications

In 2020 DOACs constituted approximately 5% of the total NHS drug budget,^43^ of which of course, could be even higher due to the pandemic of COVID. Based on the findings of our study, a health economic analysis is justified to evaluate whether the increased utilization of ED and nonelective hospitalization associated with the new use of DOAC, with their high purchasing costs of DOACs are more costly than optimized warfarin use through the adoption of novel methods such as genotype‐guided dosing^44^, ^45^ and point‐of‐Care INR monitoring.^46^ This would leave DOAC prescribing only for those at high risk of bleeding events, for example, those with variant alleles that increase the risk of bleeding from warfarin.^47^

Based on primary care electronic health record data linked with hospitalization data, via a novel tapered matching method, we formed “quasi” comparison cohorts for incident DOAC and warfarin users with AF. This study supported and extended the observations from the ECLIPSE risk stratification system (PRISM) and showed an increased risk of ED attendance, nonelective hospitalization, in new DOAC users compared with new warfarin users. For patients with AF, caution is warranted when prescribing DOACs as first‐line anticoagulant treatment. Further large‐scale replication studies involving external datasets, matching for other confounders, and over a longer period of time are warranted.

## CONFLICT OF INTEREST STATEMENT

The authors declare no conflict of interest.

## Supporting information

Supporting information.

## Data Availability

The data that support the findings of this study are available from the corresponding author upon reasonable request.
