# Supplementary figures and images for: Real‐world evidence that among atrial fibrillation patients warfarin is associated with reduced nonelective admissions compared with those on DOACs
Source: Clin Cardiol. 2023 Sep 8;46(12):1544–53. doi: 10.1002/clc.24146 (PMC10716333; doi:10.1002/clc.24146)

## Supplemental Figure-1. Workflow charts for matching process

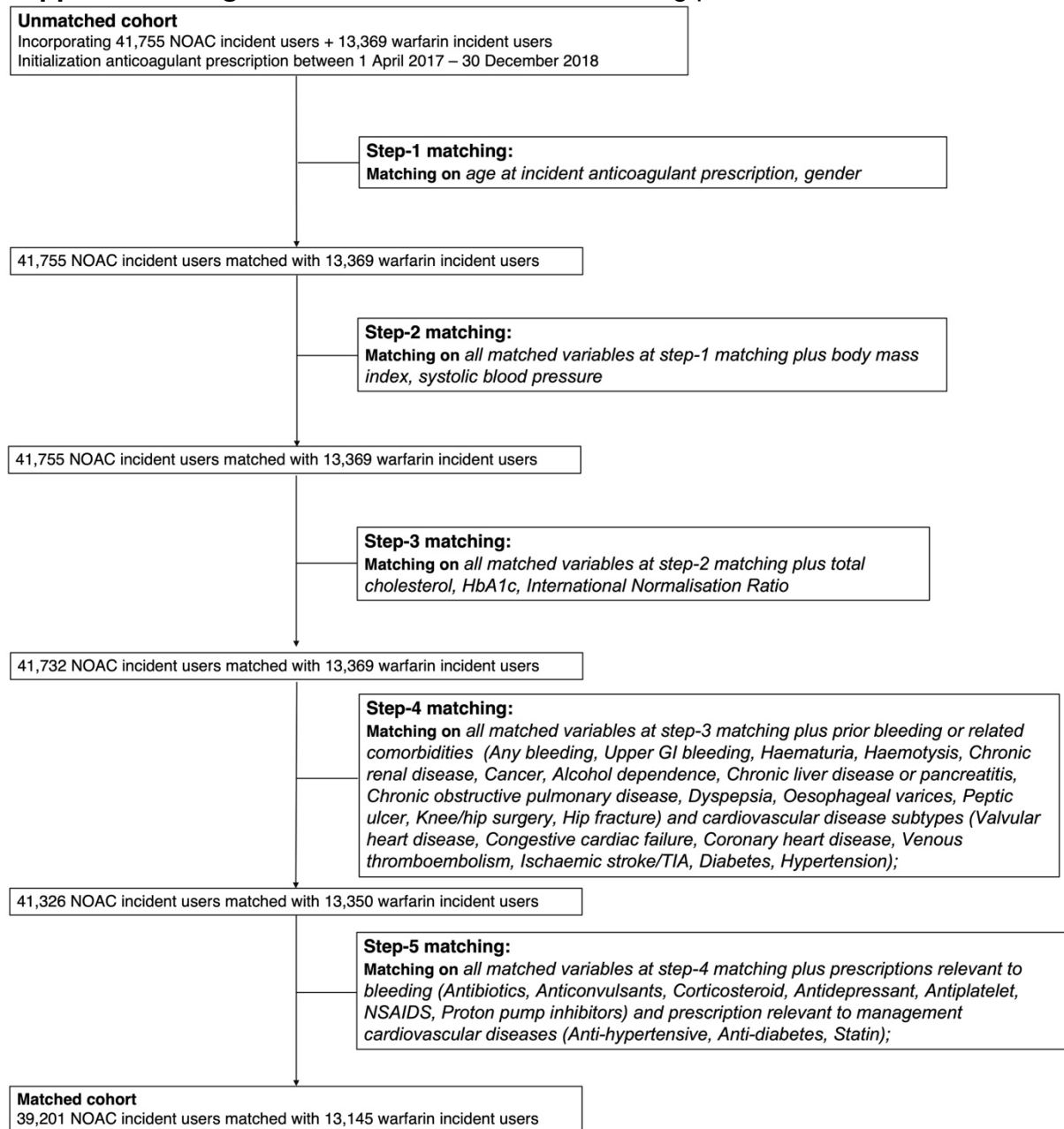

Supplement: Supplementary file 1 — Supporting information. [file CLC-46-1544-s001.pdf]
